# Fitting NTCP models to bladder doses and acute urinary symptoms during post-prostatectomy radiotherapy

**DOI:** 10.1186/s13014-018-0961-x

**Published:** 2018-02-02

**Authors:** Panayiotis Mavroidis, Kevin A. Pearlstein, John Dooley, Jasmine Sun, Srinivas Saripalli, Shiva K. Das, Andrew Z. Wang, Ronald C. Chen

**Affiliations:** Department of Radiation Oncology, University of North Carolina, 101 Manning Dr, Chapel Hill, NC 27599-7512 USA

**Keywords:** Radiobiological parameters, Acute urinary symptoms, Post-prostatectomy radiotherapy, NTCP, LKB, Logit, Relative seriality

## Abstract

**Background:**

To estimate the radiobiological parameters of three popular normal tissue complication probability (NTCP) models, which describe the dose-response relations of bladder regarding different acute urinary symptoms during post-prostatectomy radiotherapy (RT). To evaluate the goodness-of-fit and the correlation of those models with those symptoms.

**Methods:**

Ninety-three consecutive patients treated from 2010 to 2015 with post-prostatectomy image-guided intensity modulated radiotherapy (IMRT) were included in this study. Patient-reported urinary symptoms were collected pre-RT and weekly during treatment using the validated Prostate Cancer Symptom Indices (PCSI). The assessed symptoms were flow, dysuria, urgency, incontinence, frequency and nocturia using a Likert scale of 1 to 4 or 5. For this analysis, an increase by ≥2 levels in a symptom at any time during treatment compared to baseline was considered clinically significant. The dose volume histograms of the bladder were calculated. The Lyman-Kutcher-Burman (LKB), Relative Seriality (RS) and Logit NTCP models were used to fit the clinical data. The fitting of the different models was assessed through the area under the receiver operating characteristic curve (AUC), Akaike information criterion (AIC) and Odds Ratio methods.

**Results:**

For the symptoms of urinary urgency, leakage, frequency and nocturia, the derived LKB model parameters were: 1) *D*_50_ = 64.2Gy, *m* = 0.50, *n* = 1.0; 2) *D*_50_ = 95.0Gy, *m* = 0.45, *n* = 0.50; 3) *D*_50_ = 83.1Gy, *m* = 0.56, *n* = 1.00; and 4) *D*_50_ = 85.4Gy, *m* = 0.60, *n* = 1.00, respectively. The AUC values for those symptoms were 0.66, 0.58, 0.64 and 0.64, respectively. The differences in AIC between the different models were less than 2 and ranged within 0.1 and 1.3.

**Conclusions:**

Different dose metrics were correlated with the symptoms of urgency, incontinence, frequency and nocturia. The symptoms of urinary flow and dysuria were poorly associated with dose. The values of the parameters of three NTCP models were determined for bladder regarding four acute urinary symptoms. All the models could fit the clinical data equally well. The NTCP predictions of urgency showed the best correlation with the patient reported outcomes.

## Background

The knowledge of dosimetric predictors for acute genito-urinary (GU) toxicity after intensity modulated radiotherapy (IMRT) for prostate cancer is largely lacking [1–4]. There is an increasing tendency to pay more attention to quality of life (QOL) issues, which is especially relevant in prostate cancer [5]. The incidence of acute moderate/severe GU toxicities, which play a major role on QOL, has increased in recent years, due to more aggressive treatments in terms of prescribed dose and fractionation schemes [1, 2].

**Table 1:** Dosimetric parameters in the patients with and without toxicity

	No Toxicity (mean ± standard deviation)	Toxicity (mean ± standard deviation)
Frequency		
Patients	71	14
D_mean_ (Gy)	50.0 ± 11.5	47.6 ± 9.5
V18 (cc)	99.2 ± 43.3	133.4 ± 63.1
Nocturia		
Patients	71	15
D_mean_ (Gy)	49.4 ± 11.0	48.2 ± 14.4
V40 (cc)	76.7 ± 32.2	97.0 ± 48.2
Urinary urgency		
Patients	64	26
D_mean_ (Gy)	47.4 ± 11.1	53.3 ± 11.2
V18 (%)	84.2 ± 18.5	91.8 ± 15.6
Incontinence		
Patients	74	13
D_mean_ (Gy)	48.6 ± 11.9	52.4 ± 9.4
V40 (%)	68.0 ± 21.8	75.8 ± 17.8

The presented dose volume values (V_18_ and V_40_) are those that showed the highest area under the receiver operating characteristic curve (AUC) values in each case

**Table 2:** Summary of the best estimates and 95% confidence intervals of the parameters of the three examined normal tissue complication probability models for four acute bladder symptoms, respectively

Lyman-Kutcher-Burman (LKB) model				
*Symptom*	V_ref_	*D*_50_ (Gy)	*m*	*n*
Urinary urgency	Whole	64.2 (54.6–78.7)	0.50 (0.35–0.88)	1.00 (0.40–7.00)
Urinary incontinence	Whole	95.0 (77.2–130.6)	0.45 (0.35–0.62)	0.50 (0.13–3.51)
Frequency	150cm^3^	83.1 (61.3–132.9)	0.56 (0.43–0.81)	1.00 (0.55–7.00)
Nocturia	150cm^3^	85.4 (63.0–143.1)	0.60 (0.47–0.87)	1.00 (0.40–7.00)
Logit model				
*Symptom*	V_ref_	*D*_50_ (Gy)	*k*	*n*
Urinary urgency	Whole	67.6 (60.0–77.7)	3.04 (1.67–4.63)	0.49 (0.20–3.44)
Urinary incontinence	Whole	96.7 (85.8–107.6)	4.55 (3.19–5.92)	0.01 (0.01–0.07)
Frequency	150cm^3^	117.2 (82.0–183.1)	1.35 (0.95–1.86)	1.00 (0.40–7.00)
Nocturia	150cm^3^	123.1 (81.6–197.0)	1.23 (0.86–1.69)	1.00 (0.40–7.00)
Relative Seriality model				
*Symptom*	V_ref_	*D*_50_ (Gy)	γ	*s*
Urinary urgency	Whole	68.5 (55.6–86.4)	0.51 (0.28–0.74)	10^−4^ (10^−5^-7 × 10^−4^)
Urinary incontinence	Whole	103.1 (83.8–138.0)	0.59 (0.41–0.81)	0.85 (0.08–5.92)
Frequency	150cm^3^	134.7 (84.2–336.7)	0.33 (0.18–0.48)	0.82 (0.33–5.73)
Nocturia	150cm^3^	119.5 (74.7–280.9)	0.34 (0.18–0.49)	0.73 (0.29–5.12)

Responders are considered the patients with ≥2 levels of increase from baseline in the patient reported toxicity scale

**Table 3:** Summary of the results from the fit of the four normal tissue complication probability models for the different acute urinary symptoms during radiotherapy, respectively

Parameters	AUC	LL_max_	P_worse-fit_ (%)	AIC
Lyman-Kutcher-Burman (LKB) model				
Urinary urgency	0.66	−51.2	60.2	108.6
Urinary incontinence	0.58	−36.0	60.1	78.3
Frequency	0.64	−36.2	60.1	78.6
Nocturia	0.64	−38.2	60.1	82.7
Logit model				
Urinary urgency	0.66	−51.8	60.1	109.9
Urinary incontinence	0.55	−36.6	60.1	79.5
Frequency	0.64	−36.3	60.1	78.9
Nocturia	0.64	−38.2	60.1	82.8
Relative Seriality model				
Urinary urgency	0.66	−51.4	60.4	109.2
Urinary incontinence	0.57	−36.0	60.1	78.2
Frequency	0.63	−36.3	60.1	79.0
Nocturia	0.67	−37.7	60.1	81.7

*AUC* area under the receiver operating characteristic curve

*LL*_*max*_ maximum of the log-likelihood function

*P*_*worse-fit*_ probability of achieving a worse fit compared to the fitted parameter values

*AIC* Akaike information criterion

**Table 4:** Summary of the cutoff values of gEUD / $\overline{\overline{D}}$, which result in a statistically significant Odds Ratio (OR) larger than 1 for the different acute urinary symptoms

Parameters	Odds ratio	
	gEUD / $\overline{\overline{D}}$	OR (95%CI)
Lyman-Kutcher-Burman (LKB) model		
Urinary urgency	60.2	9.3 (1.7–49.8)
Urinary incontinence	60.6	3.4 (0.7–15.8)
Frequency	55.9	9.4 (1.4–62.9)
Nocturia	52.3	6.6 (1.6–27.0)
Logit model		
Urinary urgency	61.6	7.4 (1.3–40.9)
Urinary incontinence	64.9	4.1 (0.5–34.0)
Frequency	56.2	9.4 (1.4–62.9)
Nocturia	52.5	6.6 (1.6–27.0)
Relative Seriality model		
Urinary urgency	59.7	11.4 (2.2–59.7)
Urinary incontinence	60.5	3.4 (0.7–15.8)
Frequency	57.7	4.4 (1.2–16.3)
Nocturia	40.9	4.5 (1.4–14.6)

*CI* confidence interval

*gEUD* generalized Equivalent Uniform Dose

$\overline{\overline{D}}$ biologically effective uniform dose

**Fig. 1:**
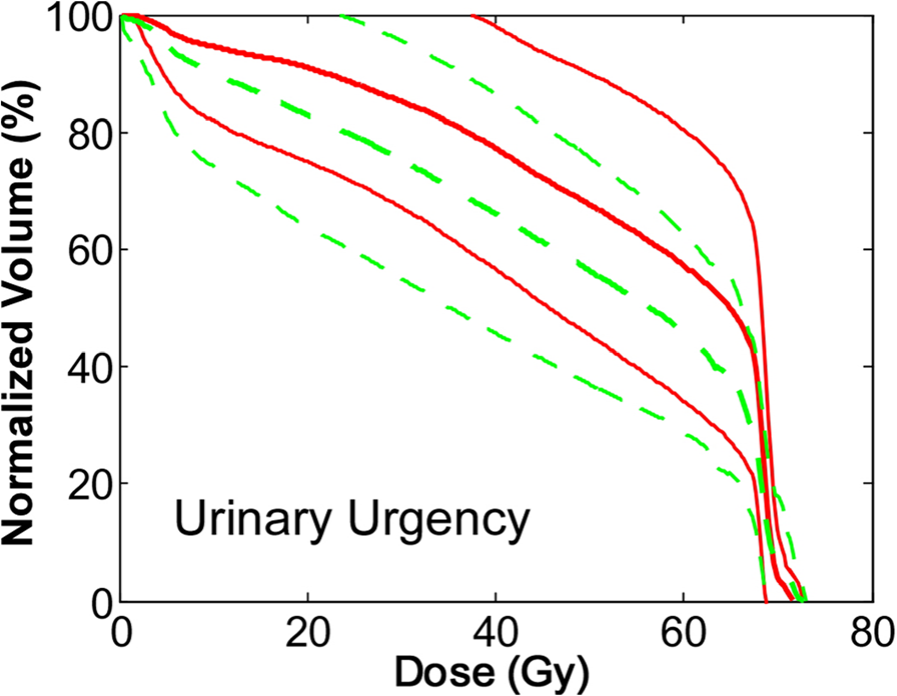
The dose-volume histograms (DVHs) of bladder for the acute symptom of urinary urgency. The DVHs of the patients with the symptom are represented by solid lines, whereas those without the symptoms by dashed lines. The thick lines represent the average DVHs, whereas the thin lines indicate the 68% confidence interval (one standard deviation)

**Fig. 2:**
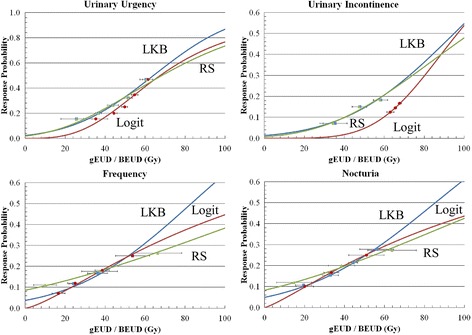
The dose-response curves of bladder, for the acute symptoms of urinary urgency, urinary incontinence, frequency and nocturia for the three models. The unit on the dose axis is either the biologically effective uniform dose ($\overline{\overline{D}}$ or BEUD), in the cases of the Relative Seriality (RS) model or the generalized equivalent uniform dose (gEUD) in the cases of the Lyman-Kutcher-Burman (LKB) and Logit models. The clinical response rates for different dose intervals (indicated by the horizontal error bars) are also plotted

Patient-reported health-related QOL is gaining recognition as an important outcome measure after cancer treatment [6–8]. The traditional approach in recording normal tissue toxicity in radiation therapy has been the use of physician-assessed scoring systems. However, there are multiple recent reports indicating that this type of assessments do not agree with patient-based reports and tend to underestimate the frequency and severity of treatment-related symptoms [9, 10]. For example, a recent study analyzed patients, who received concurrent chemoradiation therapy for rectal cancer. Although these patients showed the same grade level of diarrhea as assessed using the Common Terminology Criteria for Adverse Events (CTCAE), they had wide variations in their patient-reported diarrhea severities using a validated QOL instrument [11]. Patient-reported symptoms after different times post prostate cancer RT have been recently studied. However, this is not the case for the symptoms that develop during the course of RT treatment, involving modern intensity-modulated radiotherapy (IMRT) with image-guidance.

Modelling the relationship between dosimetric data and development of acute symptoms during treatment can ultimately lead to future radiation planning dose constraints, which may minimize these acute issues, making radiation treatment even safer and well-tolerated for patients.

At the department of Radiation Oncology of the University of North Carolina, QOL were prospectively acquired using a validated questionnaire [12] during weekly treatment visits as part of the routine clinical work-flow. The purpose of this study is to analyze dose/volume/outcome data in order to estimate the values of commonly-used NTCP models and their ability to accurately represent the data.

## Methods

### Patient data

This retrospective analysis included 93 consecutive patients, who received post-prostatectomy external beam radiation therapy (RT) between 2010 and 2015. All the patients received post-prostatectomy RT with image guidance using CT on-rails or Tomotherapy cone beam imaging. All the patients were treated with intensity modulated radiation therapy (IMRT) or volumetric modulated arc therapy (VMAT) using 6 MV photons. The prescribed dose to the planning target volume (PTV) was 66.6 Gy at 1.8 Gy per fraction delivered in 37 fractions for 7 weeks. Plans were designed to deliver at least 95% of the prescription dose to the PTV.

Patients completed the Urinary Obstruction/Irritation scale of the validated Prostate Cancer Symptom Indices (PCSI) [6, 7] before starting RT (baseline) and weekly during treatment visits. For this study seven datasets were available: 1) before or during first week of RT (93 pt); 2) second week of RT (83 pt); 3) third week of RT (84 pt); 4) forth week of RT (80 pt); 5) fifth week of RT (76 pt); 6) sixth week of RT (84 pt); and 7) seventh or last week of RT (87 pt); The patients that did not miss more than two visits and were analyzed per symptom were 88 for urine flow, 85 for frequency, 86 for nocturia, 88 for dysuria, 90 for urgency and 87 for incontinence. The symptom questions were rated on a Likert scale of 1 to 4 or 5, where 1 signified “not at all” and 4 or 5 signified “frequently or very frequently.” In this study, a score difference (maximum from all the weekly recordings) from baseline (score before RT) of ≥2 represented clinical significance [13, 14]. Six urinary symptoms were examined: 1) Urine flow; 2) Frequency; 3) Nocturia; 4) Dysuria; 5) Urgency; and 6) Incontinence. Among 93 analyzed patients, 6, 14, 15, 14, 26 and 13 had a ≥ 2 point increase in acute urinary symptoms related to urine flow, frequency, nocturia, dysuria, urgency and incontinence, respectively.

We hypothesized that bladder is the organ at risk where dose received may be associated with development of measured acute GU symptoms. Bladder delineation is part of the standard clinical practice in prostate cancer radiotherapy. The bladder dose volume histogram (DVH) was calculated for each patient from their treatment plans. These DVHs were correlated with the patient reported outcome data of different acute urinary symptoms. Figure 1 illustrates the distribution of the DVHs of the patients with and without the symptom of urgency.

### Radiobiological models

The doses in the DVHs were converted to equivalent doses of 2 Gy per fraction (EDQ_2Gy_) based on the linear quadratic model using an α/β value of 3 Gy [15, 16].

1 $$ {\mathrm{EQD}}_{2\mathrm{Gy}}=D\cdot \left(\frac{1+\frac{d}{\alpha /\beta }}{1+\frac{2}{\alpha /\beta }}\right) $$

where *D* is the physical dose, *d* is the dose per fraction. A dose distribution can be reduced to a single dose value using the generalized Equivalent Uniform Dose (gEUD) concept as follows [17, 18]:

2 $$ {\mathrm{gEUD}}_{2\mathrm{Gy}}={\left(\sum \limits_i{\left({\mathrm{EQD}}_{2\mathrm{Gy}}\right)}_i^{1/n}\frac{V_i}{V_{\mathrm{tot}}}\right)}^n $$

where *V*_*i*_ is the fractional subvolume of the organ being irradiated with a given dose and *V*_tot_ is the total volume of the organ. *n* is a parameter describing the volume dependence of the organ. gEUD_2Gy_ is then used in the LKB and Logit models as follows [19]:

3 $$ \mathrm{NTCP}=\frac{1}{\sqrt{2\pi }}\underset{-\infty }{\overset{t}{\int }}{e}^{\frac{-{x}^2}{2}} dx $$

where

4 $$ t=\frac{{\mathrm{gEUD}}_{2 Gy}-{D}_{50}}{m\cdot {D}_{50}} $$

The Logit model is a logistic equation, which produces an analytical sigmoidal shaped curve commonly used in biology and it is defined in the following way [20]:

5 $$ \mathrm{NTCP}=\frac{1}{1+{\left(\frac{D_{50}}{gEUD_{2\mathrm{Gy}}}\right)}^k} $$

The Relative Seriality (RS) model used in this work applies a different approach to account for the volume dependence and it is mathematically expressed as follows [21, 22]:

6 $$ \mathrm{NTCP}={\left[1-\prod \limits_{i=1}^M{\left(1-P{\left({D}_i\right)}^s\right)}^{\Delta {v}_i}\right]}^{1/s} $$

where

7 $$ P\left({D}_i\right)=\exp \left[-{e}^{e\gamma -\left({EQD}_{2\mathrm{Gy}}^i/{D}_{50}\right)\cdot \left( e\gamma -\ln \ln 2\right)}\right] $$

where *P*(*D*_*i*_) is the probability of response of an organ having the reference volume and being irradiated to dose *D*_*i*_. From the NTCP values the biologically effective uniform dose ($\overline{\overline{D}}$) can be derived by the following formula:

8 $$ P\left(\overrightarrow{D}\right)\equiv P\left(\overline{\overline{D}}\right)\Rightarrow \overline{\overline{D}}=\frac{e\gamma -\ln \left(-\ln \left(P\left(\overrightarrow{D}\right)\right)\right)}{e\gamma -\ln \left(\ln 2\right)} $$

The basic parameters of each model are: *D*_50_, which is the dose for a complication rate of 50%, the slope (gradient) of the dose response curve (*m* for LKB, *k* for Logit and γ for RS), and the parameter that accounts for the volume dependence of the organ (*n* for LKB and Logit, *s* for RS).

### Statistical methods for fitting the NTCP models and evaluating the goodness-of-fit

The values of the parameters of the NTCP models and their 95% confidence intervals were determined using the maximum likelihood method. In this process, the predictions of the NTCP models were fitted to the clinical outcome results by changing the values of the model parameters until best estimates could be reached [23, 24]. The profile likelihood method was used to determine the confidence intervals of the model parameters. This method is often used when accurate interval estimates are difficult to obtain using standard methods (for example, when the log-likelihood function is highly non-normal in shape). For the 95% confidence region, the allowable difference from the maximum $\ln {\overline{L}}_{\mathrm{max}}\left({\overline{x}}_i\right)-\ln {L}_{\mathrm{max}}\left({x}_i\right)$ is 1/2 × 3.84 = 1.92, for one degree of freedom.

The goodness-of-fit of the different NTCP models was assessed through the area under the receiver operating characteristic curve (AUC), maximum of the log-likelihood function, normal error distribution and Akaike information criterion (AIC) [25, 26]. More specifically, the AUC of a receiver operating characteristic (ROC) curve was computed and compared to the level of 0.5 (equivalent to a random predictor) [26]. The probability of achieving a worse fit was assessed by comparing the maximum log-likelihood value against the average log-likelihood value and its variance assuming a Gaussian distribution of the log-likelihood function [14, 23]. The Akaike information criterion was used to compare the fitting and complexity of the different models [25]. A lower Akaike number for a model indicates superiority of that model. Finally, the Odds Ratio (OR) method was applied to identify NTCP thresholds beyond which the risk of toxicity increases significantly.

## Results

Table 1 presents a summary of the average mean doses to bladder for the patients with and without acute urinary symptoms. Additionally, the average values of the dose-volume metrics that correlated best with the outcome date are shown. Urine flow and dysuria were not included in this table because the correlation between their dosimetric and outcome data was poor. Consequently, the values of the parameters of the NTCP models were determined for the symptoms of urgency, incontinence, frequency and nocturia (Table 2). The corresponding dose-response curves for the examined models and structures are shown in Fig. 2 Broadly, speaking, the three examined models essentially show equivalent goodness-of-fit per symptom. The analytical results are shown in Table 3. For the models and symptoms shown in this table, statistically significant ORs were identified for a given gEUD or $\overline{\overline{D}}$ cutoffs in every case (Table 4). More specifically, for the symptom of urgency, the biological doses range between 59.7–61.6 for corresponding statistically significant ORs of 7.4–11.4. For frequency, the biological doses range between 55.9–57.7 for ORs of 4.4–9.4. For nocturia, the biological doses range between 40.9–52.5 for ORs of 4.5–6.6. For the symptom of incontinence, no statistically significant dose cutoffs and OR were found. The NTCP predictions against the actual response rates were in agreement (differences ranged between 0.0–0.1%) for all the models and symptoms. Regarding the correlation of the NTCP prediction against the actual outcome data, the symptom of urgency shows slightly higher AUC values (0.66), but worse AIC values (108.6–109.9). On the other hand, the symptom of frequency shows the best combination of AUC and AIC values (0.63–0.64 and 78.6–79.0, respectively) (Table 3).

## Discussion

It is well-known that radiation therapy causes an impact on QOL and specifically patient-reported urinary issues. Many patients after radical prostatectomy require radiation treatment to the prostate bed; these patients are especially vulnerable to urinary symptoms during radiation treatment due to the prior surgery. However, the association between doses received to the bladder and development of patient-reported acute urinary issues in this specific patient population has not been well-studied. To our knowledge, this is the first study to address this important knowledge gap.

Many of these studies use physician-reported toxicity, but patient-reported outcomes (PRO) have become an increasingly important way to measure quality of life (QOL) after cancer treatment [6–10]. Reports indicate that PRO-QOL data are often not in-line with physician-reported acute toxicity for prostate cancer patients [13, 27–32]. Additionally, the acute toxicity of post-prostatectomy radiation and dosimetric correlations continues to be understudied. Although, there are many studies investigating frequency and severity issues of acute toxicity after prostate cancer radiotherapy, they are mostly based on physician-reported measures of quality-of-life [13, 27–38]. Additionally, not all patients treated in these studies were treated using modern treatment techniques. All the patients included in this study were treated with IMRT receiving a median dose of 66.6 Gy. They also underwent regular image guidance. This represents modern-era treatment techniques for this cohort, and should therefore provide an accurate picture of acute GU toxicity in current practice.

The model parameters based on the LENT/SOMA scoring system (≥ Grade 2) were *D*_50_ = 69.56 Gy, γ = 1.7 and *s* = 0.35 for the RS model and *D*_50_ = 78.68 Gy, *m* = 0.17 and *n* = 0.09 for the LKB model [39, 40]. Regarding the symptoms of bladder contracture and volume loss, the parameters of the RS model are *D*_50_ = 80.0 Gy, γ = 3.0 and *s* = 0.18, whereas those of the LKB model are *D*_50_ = 80.0 Gy, *m* = 0.11 and *n* = 0.5 [41–43]. All the aforementioned parameters were derived using physician-rated outcome scores. The last few years, there is a trend to evaluate radiation-induced toxicity using patient-reported outcome scores. For the symptom of urgency, the parameters for the LKB model are *D*_50_ = 150.0, *m* = 0.37 and *n* = 0.01 [44]. The values that were derived by the present study using a different outcome scoring system for the symptom of urgency during the period of RT indicate a higher radiosensitivity (lower *D*_50_). Regarding acute urinary symptoms, it has been recently reported that an impact of dosimetric parameters was found for most symptoms (frequency, intermittency, urgency and nocturia), confirming the existence of a dose–volume/surface effect for acute effects [45]. However, those data and similar ones from other studies have not been modelled yet. So, in the literature there is lack of model parameter values for acute urinary symptoms based on PRO-QOL scoring protocols.

As indicated by the results of the goodness-of-fit, the three models studied here fitted the clinical data with similar accuracy. However, it should be pointed out that an agreement between those models is observed when fitting organs showing a parallel behavior, whereas discrepancies between them are observed when fitting organs of serial behavior. In the literature, we could not identify any other study deriving parameters for multiple NTCP models for acute urinary symptoms. In order to clinically validate the derived parameters, an independent cohort of patients with similar clinical characteristics should be available. In this study, the correlation of those parameters with the outcome data was mainly performed to identify the differences between the models regarding the goodness-of-fit process.

It should be noticed that for the symptoms of urgency and incontinence, the dose/volume metrics that correlated with the outcome data and the NTCP models had the volume expressed in percentage of the whole of volume of bladder, whereas frequency and nocturia had the best correlation when the volume was expressed in absolute units (cc). This means that for the symptoms of frequency and nocturia it is not the relative size of bladder receiving a given dose that is associated with them but the absolute volume of bladder. It is also clinically relevant that we did not find a clear association between bladder dose and urinary flow or dysuria. This is consistent with our clinical hypotheses. Radiation therapy can cause slower urinary flow due to swelling of the prostate which obstructs the urethra; but in patients who have previously undergone a radical prostatectomy, this mechanism does not apply. Further, clinically, dysuria is commonly deemed to be related to radiation irritation of the urethra, which was not assessed in this study because a Foley catheter is not routinely used in our treatment planning process. Therefore, the null findings in our analysis related to urine flow and dysuria provide further face validity regarding the results.

The current study does not account for other clinical factors that may have been associated with acute urinary toxicity, such as the use of medications (hormonal therapy), Prostate-specific antigen (PSA), Gleason score and volume of the gross tumor volume (GTV). However, few of these factors have consistently been found to impact acute toxicity [1, 46]. Most of those factors are not directly related to the function of bladder but mostly affect the treatment plan and consequently the dose distribution delivered to the patient. So, analyzing the dose to bladder against those symptoms should lead to better correlations. Moreover, given the small sample size of this study additional subset analyses could not performed.

A few points of caution should be mentioned regarding this study. First, it is difficult to compare the results of this study with previously reported findings due to the fact that a new patient reported outcome system (PCSI) was used for the acute urinary symptoms instead of the most common CTCAE scoring system. Second, although patient toxicity was based on the validated QOL instrument specifications, the definition of a significant toxicity was somewhat subjective (a two-point increase in the symptom score on the four-point symptom scale). However, other groups using patient reported outcomes have followed this approach because it acknowledges the importance of taking into account the baseline status [47–49]. Third, we had limited possibility of performing sub-sample test validation or k-fold validation of our models due to the size of our dataset. A future goal should be to collect new clinical data to separately validate the predictive ability of the models and their suitability to be used as constraints in treatment planning.

## Conclusions

In this study it was shown that all the examined NTCP models (LKB, Logit and Relative Seriality) could fit the individual patient reported outcome data with very similar accuracy. The NTCP predictions of frequency correlated a little better with the outcome data than the rest of the acute symptoms. The values of the model parameters for the different acute symptoms could not be compared with previously published values, which is most likely attributed to the patient reported outcome system used instead of the CTCAE system. Further investigation with a larger patient cohort could verify the suitability of using the values of the NTCP model as an additional constraint in IMRT treatment plan optimization.

## Abbreviations

$\overline{\overline{D}}$ or BEUD: biologically effective uniform dose
AIC: Akaike information criterion
AUC: area under the curve
CTCAE: Common Terminology Criteria for Adverse Events
DVH: dose volume histogram
EDQ_2Gy_: equivalent doses of 2 Gy per fraction
gEUD: generalized Equivalent Uniform Dose
GTV: gross tumor volume
GU: genito-urinary
IMRT: intensity modulated radiotherapy
LKB: Lyman-Kutcher-Burman
NTCP: normal tissue complication probability
OR: Odds Ratio
PCSI: Prostate Cancer Symptom Indices
PRO: patient-reported outcomes
PSA: Prostate-specific antigen
PTV: planning target volume
QOL: quality of life
ROC: receiver operating characteristic
RS: Relative Seriality
RT: radiation therapy
VMAT: volumetric modulated arc therapy
